# Mortality risk of carbapenem-resistant hypervirulent *Klebsiella pneumoniae* vs. classical CR-KP: a systematic review and meta-analysis

**DOI:** 10.3389/fpubh.2025.1680292

**Published:** 2025-11-06

**Authors:** Jialiang Chen, Yue Hou, Jingang Shi, Xiaoyi Gao, Guowei Liang

**Affiliations:** 1 Department of Clinical Laboratory, Aerospace Center Hospital, Beijing, China; 2 Translational Medicine Center, Beijing Chest Hospital, Capital Medical University, Beijing, China; 3 Institute of Tuberculosis and HIV/AIDS Control and Prevention Hohhot Center for Disease Control and Prevention, Hohhot, China

**Keywords:** *Klebsiella pneumoniae*, carbapenem resistance, hypervirulence, CR-hvKP, dual-threat pathogens, ST11 clone, infection outcomes, mortality

## Abstract

**Background:**

Carbapenem-resistant *Klebsiella pneumoniae* (*K. pneumoniae*, CR-KP) and hypervirulent *K. pneumoniae* (hvKP) represent two major clinical threats, due to high antimicrobial resistance and enhanced pathogenicity, respectively. The emergence of carbapenem-resistant hypervirulent *K. pneumoniae* (CR-hvKP) strains, which combine both traits, has raised concerns about increased mortality risk and public health impact. However, existing evidence on clinical outcomes remains fragmented and inconclusive. To systematically compare the mortality risk between CR-hvKP and classical CR-KP (CR-cKP) infections and to explore the impact of hypervirulence definitions, mortality endpoints, infection types, and clinical settings through subgroup analyses.

**Methods:**

We conducted a comprehensive literature search in PubMed, Scopus, Web of Science, and EMBASE up to July 1, 2025. Studies reporting mortality outcomes in patients infected with CR-hvKP and CR-cKP were included. Pooled odds ratios (ORs) with 95% confidence intervals (CIs) were calculated using a random-effects model. Subgroup analyses were performed to investigate heterogeneity. Sensitivity analysis and Egger’s regression test were used to assess robustness and publication bias.

**Results:**

Ten studies with a total of 770 patients (224 with CR-hvKP, 546 with CR-cKP) were included, reporting 315 deaths. The pooled OR for mortality associated with CR-hvKP infection was 2.05 (95% CI: 0.89–4.75), indicating a non-significant trend toward higher mortality. Subgroup analyses indicated significantly increased mortality in studies using phenotypic string tests to define hypervirulence (OR = 4.16), but not in those using genotypic definitions (OR = 1.05). Higher mortality trends were also observed for in-hospital mortality, bloodstream infections, and ICU settings.

**Conclusion:**

CR-hvKP may be associated with higher mortality risk compared to CR-cKP. The heterogeneity in hypervirulence definitions significantly influences outcome estimates, highlighting the urgent need for standardized diagnostic criteria. These findings underscore the importance of ongoing molecular surveillance, early identification strategies, and targeted infection control measures to mitigate the public health threat posed by CR-hvKP.

**Systematic review registration:**

https://www.crd.york.ac.uk/PROSPERO/view/CRD420251117975, identifier CRD420251117975.

## 1 Introduction

*Klebsiella pneumoniae* (*K. pneumoniae*), a Gram-negative opportunistic pathogen, is a major cause of nosocomial infections and can lead to a wide range of clinical diseases, including pneumonia, urinary tract infections, and bloodstream infections (1, 2). According to data from the China Antimicrobial Resistance Surveillance System (CARSS) in 2022, *K. pneumoniae* ranked second among Gram-negative bacteria in China, with an isolation rate of 21.1%, and showed a continuously increasing trend. Of particular concern is the dissemination of multidrug-resistant strains carrying extended-spectrum *β*-lactamases (ESBLs) and carbapenemases, which has made carbapenem-resistant *K. pneumoniae* (CR-KP) a significant global health threat, severely limiting therapeutic options. Although CR-KP and hvKP have been reported worldwide, epidemiological patterns show marked regional variation, with a predominance of data from Asia. At the same time, hypervirulent *K. pneumoniae* (hvKP) has emerged as an important cause of severe community-acquired infections. The hvKP, first identified in 1986 in Taiwan, China, has since become globally recognized for its high pathogenicity and distinct phenotypic and genotypic characteristics (3). It can cause invasive infections even in healthy individuals, including liver abscesses (4, 5), pulmonary infections (6), and meningitis (7). Currently, there is no consensus on the definition of hvKP. Some studies define it based on the presence of virulence genes such as *rmpA*, *iucA*, and *iroB*, while others consider phenotypic characteristics such as hypermucoviscosity and excessive capsule production as indicators of high virulence (8).

Traditionally, it has been thought that there is a trade-off between antibiotic resistance and virulence (9–11), where CR-KP is typically associated with high resistance but lower virulence, whereas hvKP is highly virulent but generally susceptible to antimicrobials (12). However, the widespread use of carbapenems has accelerated plasmid-mediated horizontal gene transfer, giving rise to so-called “dual-threat” pathogens—carbapenem-resistant hypervirulent *K. pneumoniae* (CR-hvKP)—that combine both resistance and hypervirulence. These strains may arise through (a) acquisition of carbapenemase genes by hvKP; (b) acquisition of virulence plasmids by CR-KP; or (c) uptake of hybrid plasmids carrying both resistance and virulence determinants (13). The emergence of CR-hvKP challenges the previous notion that resistance and virulence are mutually exclusive, and poses significant clinical risks, including invasive infections, treatment failure, and increased mortality (14). CR-hvKP has already been reported in many parts of the world, with Asia being particularly affected (15). In a tertiary hospital in northern China, molecular epidemiological analysis of 100 CR-KP strains from 2016 to 2023 revealed that 66% were CR-hvKP (16). Another study from a Chinese tertiary hospital analyzing 2,002 *K. pneumoniae* isolates collected between 2014 and 2018 found that CR-hvKP accounted for all deaths, with an infection mortality rate of 20%, and the highest mortality observed in bloodstream infections (17). However, most available epidemiological evidence to date originates from East Asia, particularly China, while data from Europe, the Americas, and Africa remain scarce. This geographic concentration highlights a major epidemiological gap and may limit the global generalizability of current findings. Recognizing this limitation is essential for interpreting pooled evidence and for identifying priorities for future multinational research. These findings underscore the growing prevalence and clinical burden of CR-hvKP.

Although several studies have investigated the clinical outcomes of CR-hvKP infections, their conclusions vary significantly due to heterogeneity in virulence definitions (e.g., gene detection, string test, animal models), types of virulence genes analyzed (e.g., regulator of mucoid phenotype, mucoviscosity-associated gene), sources of isolates (community-acquired, hospital-acquired), patient departments (ICU, internal medicine, surgery), infection sites (bloodstream, respiratory tract, urinary tract), and outcome definitions (e.g., 30-day mortality, in-hospital mortality). To date, no comprehensive quantitative analysis has systematically compared the all-cause mortality between CR-hvKP and CR-cKP infections.

Given the growing threat of CR-hvKP and the fragmented, heterogeneous evidence available, it is both necessary and urgent to conduct a systematic review and meta-analysis comparing the mortality risk between CR-hvKP and CR-cKP infections. This study aims to integrate existing clinical evidence, quantitatively evaluate the difference in mortality between the two groups by calculating pooled odds ratios (ORs), and explore the impact of different detection methods, infection types, geographic regions, and outcome definitions through subgroup analyses. Sensitivity analyses will be performed to assess the robustness of the findings. The results of this meta-analysis will not only provide an evidence-based understanding of the clinical outcomes of CR-hvKP infections but may also contribute to the standardization of future CR-hvKP research methods and support the development of targeted clinical management strategies. Additionally, it will offer valuable theoretical and epidemiological insights for molecular surveillance and risk assessment of hypervirulent, multidrug-resistant pathogens.

## 2 Materials and methods

The systematic review and meta-analysis followed a protocol registered with PROSPERO (CRD420251117975) and adhered to the Preferred Reporting Items for Systematic Reviews and Meta-Analyses (PRISMA) guidelines.

### 2.1 Search strategy

We systematically searched electronic databases, including PubMed, Scopus, Web of Science, and EMBASE until July 1, 2025, using suitable keywords. No language or date restrictions were used. The full strategy for each database is illustrated in Supplementary Table S1. All retrieved studies were imported into EndNote X9 for duplicate removal. Two trained researchers independently screened titles and abstracts to exclude studies not fulfilling the inclusion criteria. Subsequently, two independent reviewers will assess the full texts and discard ineligible studies. Disagreements at either stage will be resolved by a third researcher. Studies were included if only a subgroup satisfied the eligibility criteria, with solely subgroup-level data extracted.

### 2.2 Eligibility criteria

Studies were included if they clearly distinguished between CR-hvKP and CR-cKP and reported mortality outcomes. Given the absence of a universal diagnostic standard for hvKP, studies included in this meta-analysis used either genotypic (virulence gene detection) or phenotypic (string test) definitions. This inherent heterogeneity in defining hypervirulence represents an *a priori* limitation of our analysis, as these differing criteria may capture distinct bacterial populations. Thus, the definition is based on either: (a) molecular detection of virulence genes (e.g., *rmpA*, *rmpA2*, *iucA*, *iroB*, *peg-344*, with the positive result defined as hvKP), (b) phenotypic assays (e.g., string test, with the positive result defined as hypermucoviscosity), or explicit author classification. Accordingly, the CR-cKP group in each study comprised carbapenem-resistant isolates that were negative for the specific hypervirulence definition applied in that study. Eligible study designs included prospective cohort studies, retrospective cohort studies, case–control studies, and cross-sectional studies. The primary outcome was mortality (all-cause or infection-related mortality), including: (a) short-term (e.g., 14-day, 28-day, 30-day) or in-hospital mortality, and (b) effect measures (e.g., ORs, HRs, adjusted or crude) for CR-hvKP versus CR-cKP.

**Figure 1:**
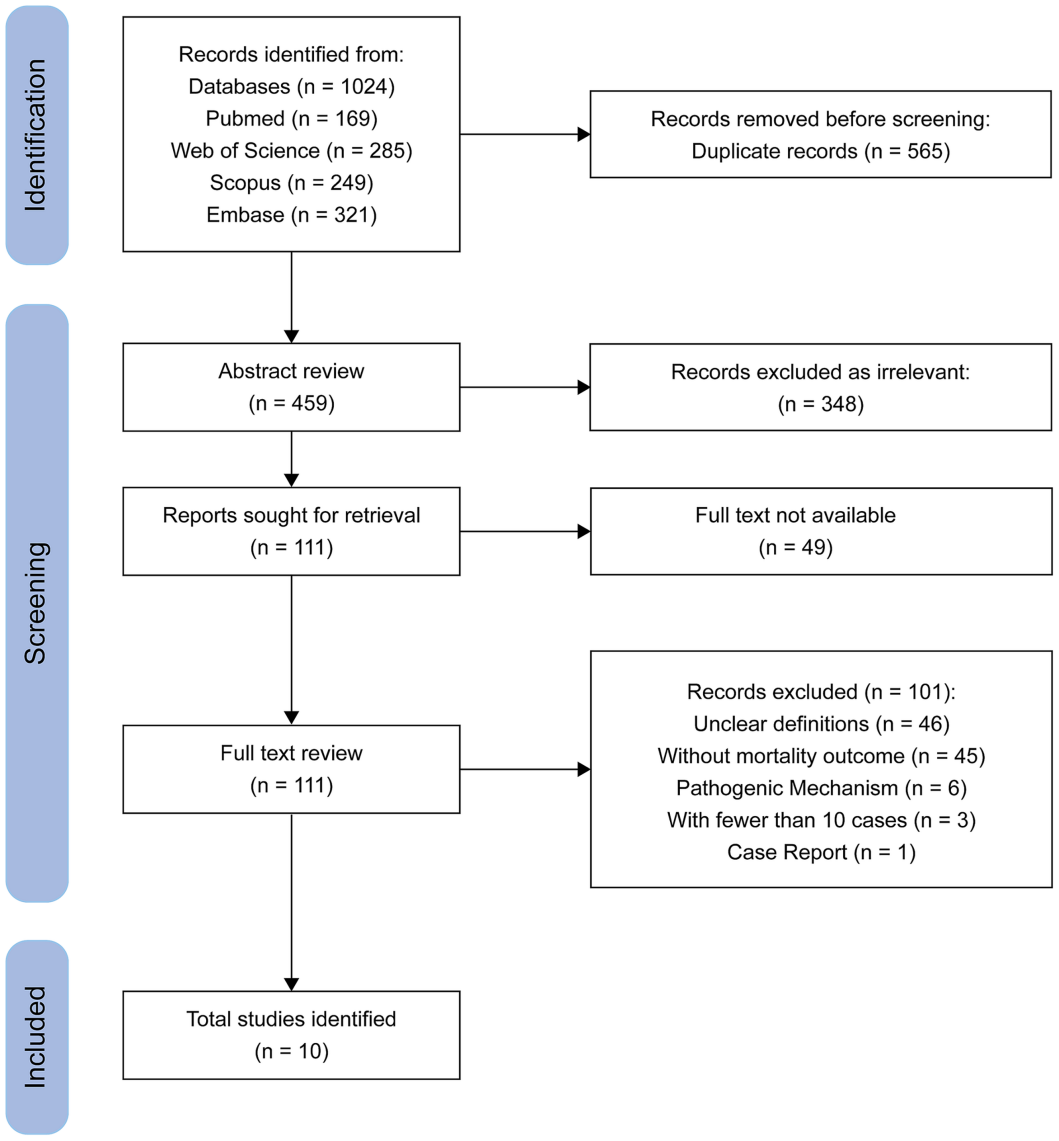
PRISMA flowchart for systematic review and meta-analysis.

Reviews, comments, conference abstracts, case reports, and mechanistic studies without clinical data, animal studies, and studies involving environmental isolates were excluded. Studies were also excluded if they did not clearly distinguish between CR-hvKP and CR-cKP, or if they did not report mortality outcomes. For studies using overlapping or duplicate datasets, only the most comprehensive or most recent publication was included to ensure non-redundant data. Studies involving fewer than 10 combined cases in both groups were excluded to minimize instability of effect estimates and reduce potential bias. A total of three studies were excluded on this basis, as shown in the PRISMA flow diagram (Figure 1).

### 2.3 Data extraction and quality assessment

Data extraction was performed independently by two reviewers, with disagreement resolved through consensus or arbitration. The following data were extracted: basic information (including author, country, and year of publication); study characteristics (including number of patients, gender, age, hvKP identification method, and infection type); and outcome data (such as mortality, number of cases in the CR-hvKP and CR-cKP groups, and timing of events). All data were obtained from original publications or the publicly available Supplementary Table S2. Additionally, most included studies did not provide detailed patient-level information, such as comorbidities, immunosuppression status, or antimicrobial treatment regimens. The absence of these variables precludes adjustment for potential confounders, and residual confounding is therefore likely, which may bias the comparison of mortality outcomes between CR-hvKP and CR-cKP.

Study quality was assessed using the Agency for Healthcare Research and Quality (AHRQ) criteria for observational studies, which comprise 11 items scored as “yes” (1 point), “no” (0 points), or “unclear” (0 points), with a total possible score of 11. A score of 0–3 was considered low quality, 4–7 moderate quality, and 8–11 high quality. Studies with low quality (score < 4) were excluded. In cases of disagreement, a third reviewer was consulted to reach a consensus. Quality assessment scores for each study are presented in Supplementary Table S3.

### 2.4 Data synthesis and statistical analysis

Analysis was performed using R software (version 4.3.1). ORs with 95% confidence intervals (CIs) for mortality were calculated based on extracted counts. A random-effects meta-analysis was conducted using the DerSimonian-Laird estimator via the “meta” package. Between-study heterogeneity was assessed using the I^2^ statistic; an I^2^ value greater than 50% combined with a *p* < 0.05 was considered indicative of significant heterogeneity. Subgroup analyses were performed based on hypervirulence detection method (virulence gene detection and string test), infection site (bloodstream and non-bloodstream), and mortality timepoints (in-hospital, 14-day, and 30-day). To explore potential sources of heterogeneity, meta-regression was conducted using the “metafor” package, with the median strain collection year (i.e., the median year of strain collection) included as a covariate. Sensitivity analyses were conducted by sequentially omitting individual studies to assess the robustness of the findings. Publication bias was assessed visually using funnel plots and statistically using Egger’s regression test for funnel plot asymmetry. The Egger test was conducted using the metabias function in the “meta” package, with a significance threshold of *p* < 0.05.

## 3 Results

### 3.1 Results of literature search and screening

The initial search yielded 1,024 publications. After removing 565 duplicate records, 348 studies were excluded based on title and abstract screening. Full-text assessment was conducted for the remaining 111 articles, of which 101 were excluded for various reasons. Ultimately, 10 studies met the inclusion criteria and were included in the final analysis. A flow diagram of the literature search and screening process is presented in Figure 1.

### 3.2 Characteristics of included studies and risk of bias assessment

The characteristics of the included studies and the results of the risk of bias assessments are summarized. This meta-analysis included 10 studies comprising a total of 770 patients (224 with CR-hvKP and 546 with CR-cKP). Across these studies, a total of 315 mortality events were reported (Table 1).

**Table 1:** Key characteristics of included studies.

Study	Median sampling year	Depart-ment	Infection type	hvKP definition standard*	Event/Total of CR-hvKP	Event/Total of CR-cKP	AHRQ	Mortality timepoints
Du et al. (39)	2017	ICU	Bloodstream	S	13/14	15/26	6	In-hospital
Lei et al. (27)	2020	ICU	Other	V	5/7	9/42	5	In-hospital
Li et al. (40)	2016	other	Bloodstream	V	11/20	34/64	6	30-day
Ouyang et al. (30)	2018	other	Other	V	7/41	5/21	5	In-hospital
Pan et al. (41)	2014	NA	Other	S	9/15	29/51	5	In-hospital
Shankar et al. (20)	2015	ICU	Bloodstream	S	20/27	29/59	5	30-day
Wei et al. (28)	2020	other	Other	V	21/51	15/29	6	In-hospital
Xu et al. (42)	2014	other	Bloodstream	S	3/3	39/92	5	14-day
Yang et al. (31)	2019	other	Other	V	10/31	11/31	6	30-day
Zhang et al. (22)	2018	other	Bloodstream	S	10/15	20/131	6	In-hospital

^*^S, string test; V, virulence genes test; NA, Unknown.

Geographically, nine studies were conducted in China and one in India. The strain collection periods spanned from 2013 to 2021. Five studies focused specifically on bloodstream infections, while the remaining included patients with mixed infection types, such as respiratory tract infections, urinary tract infections, or skin and soft tissue infections. Patient characteristics varied across studies. In all studies, the proportion of male patients exceeded that of females, although the exact ratios differed. Age distributions also varied, reflecting differences in patient selection and inclusion criteria.

Definitions of hvKP were heterogeneous. Some studies used genotypic criteria, identifying hvKP based on the presence of virulence-associated genes such as *rmpA*, *iucA*, *iroB*, and *peg-344*. Others adopted a phenotypic definition using the string test, with >5 mm mucoviscosity considered positive. One study initially incorporated the animal infection model to define hypervirulence; however, due to the lack of standardized criteria and the inconsistency with definitions used in other studies, this study was excluded from the final meta-analysis.

The quality of the included studies was assessed using the 11-item AHRQ checklist for cross-sectional studies. Scores ranged from 5 to 6 out of a maximum of 11, with a mean score of 5.5, indicating overall moderate methodological quality. All studies clearly defined the source of data, inclusion and exclusion criteria, and the period used for case identification. However, none of the studies clarified whether patient enrollment was consecutive, and subjective components were not reported as being masked. Common limitations included lack of control for potential confounders, inadequate handling of missing data, and absence of response rate reporting. A summary of the AHRQ domain-level scoring is provided in Table 1.

### 3.3 Total mortality

**Figure 2:**
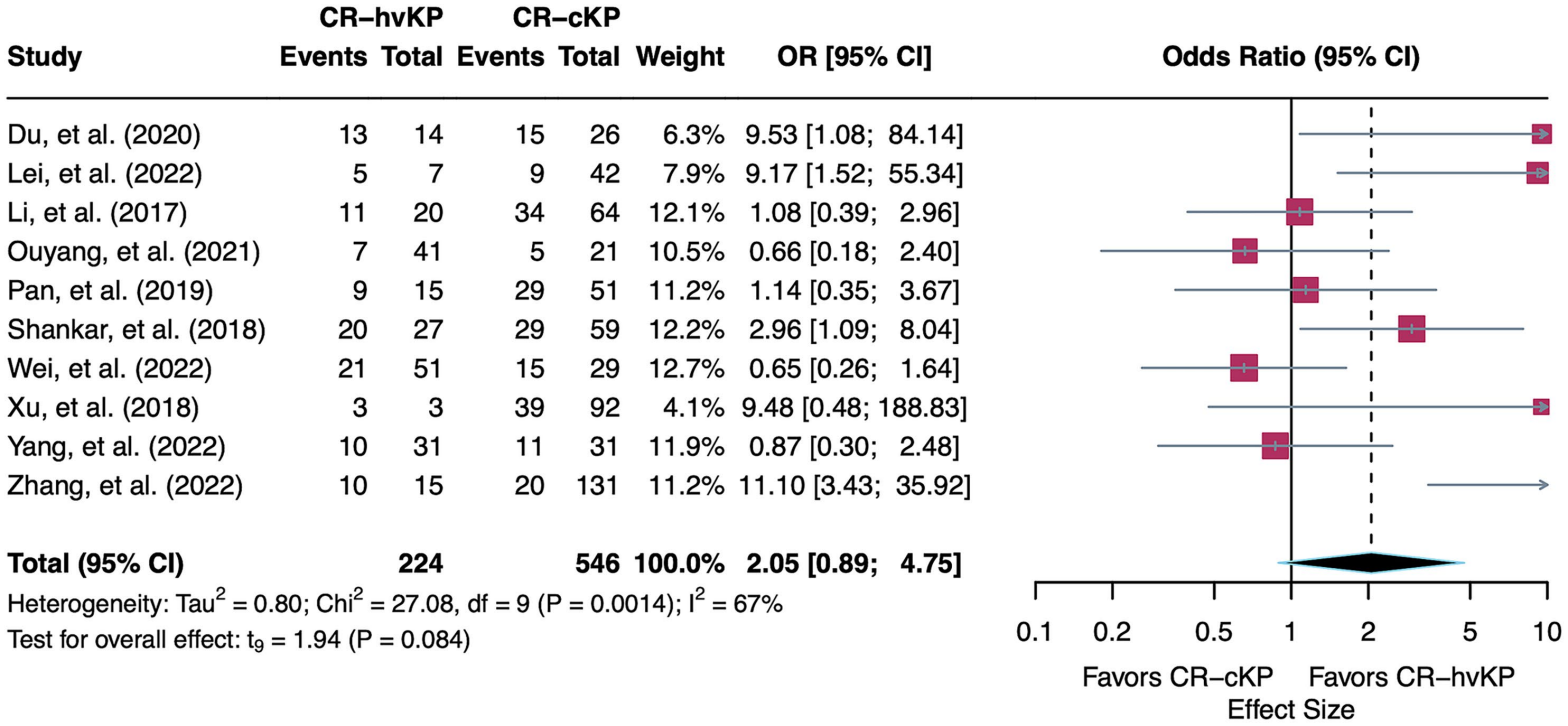
Forest plot showing the pooled OR for mortality comparing CR-hvKP and CR-cKP. ORs > 1 indicate higher mortality in CR-hvKP compared to CR-cKP, while ORs < 1 indicate higher mortality in CR-cKP.

A total of 10 studies were included in the meta-analysis comparing mortality between patients infected with CR-hvKP and with CR-cKP. Using a random-effects model, the pooled OR was 2.05 (95% CI: 0.89–4.75, *p* = 0.08), indicating a non-significant trend toward higher mortality in the CR-hvKP group. This association should be interpreted cautiously, given the wide confidence interval and lack of statistical significance. Moderate heterogeneity was observed across the included studies (I^2^ = 66.8%, τ^2^ = 0.80, Q = 27.08, *p* < 0.01) (Figure 2).

To investigate sources of heterogeneity, prespecified subgroup analyses were performed based on hypervirulence identification method, mortality timepoint, infection type, and patient department. In addition, meta-regression was conducted to assess the potential influence of sampling year.

### 3.4 Subgroup analyses

**Figure 3:**
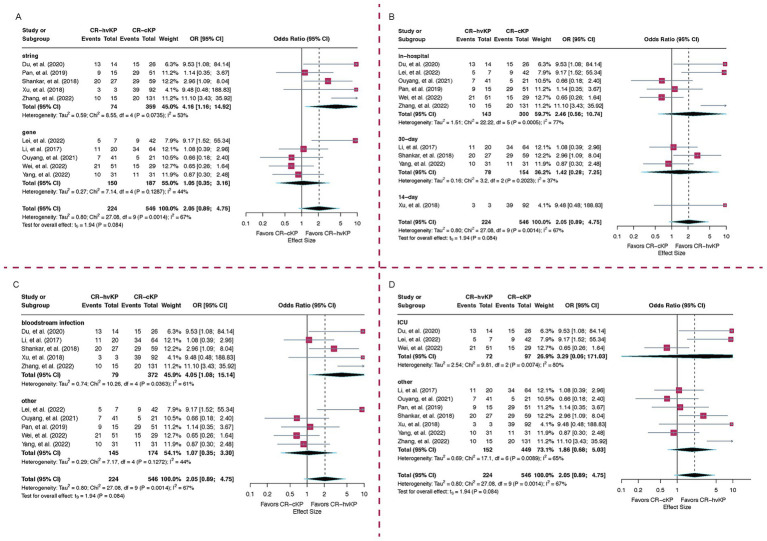
Subgroup analyses of mortality risk comparing CR-hvKP and CR-cKP. **(A)** Based on hypervirulence detection methods, **(B)** Based on mortality timepoints, **(C)** By infection type, **(D)** By hospital department. ORs > 1 indicate higher mortality in CR-hvKP compared to CR-cKP, while ORs < 1 indicate higher mortality in CR-cKP.

Subgroup analysis by hypervirulence detection method revealed that studies using genotypic criteria (*n =* 5) showed no significant association between CR-hvKP and increased mortality (OR = 1.05, 95% CI: 0.35–3.16, I^2^ = 44.0%). In contrast, studies employing the string test (*n =* 5) demonstrated a significantly elevated mortality in the CR-hvKP group (OR = 4.16, 95% CI: 1.16–14.92, I^2^ = 53.2%). The between-group difference was statistically significant (*p* = 0.02), suggesting that the method used to define hypervirulence may substantially influence the observed effect. Notably, the heterogeneity decreased to 44.0% following stratification by detection method, indicating that this variable contributed to between-study variability (Figure 3A).

Regarding mortality timepoints, six studies reported in-hospital mortality, with a pooled OR of 2.46 (95% CI: 0.56–10.74, I^2^ = 77.5%). Three studies assessed 30-day mortality (OR = 1.42, 95% CI: 0.28–7.25, I^2^ = 37.4%), while one study reported 14-day mortality (OR = 9.48, 95% CI: 0.48–188.83). The between-group difference in time-limited mortality was not statistically significant (*p* = 0.39). These findings highlight variability in mortality definitions and suggest that short-term endpoints may underestimate the impact of CR-hvKP (Figure 3B).

When stratified by infection type, patients with bloodstream infections (*n =* 5) had a significantly higher mortality associated with CR-hvKP (OR = 4.05, 95% CI: 1.08–15.14, I^2^ = 61.0%), whereas studies focusing on non-bloodstream infections (*n =* 5) showed no significant difference (OR = 1.07, 95% CI: 0.35–3.30, I^2^ = 44.2%). A statistically significant trend (*p* = 0.04) toward higher mortality was observed in patients with bloodstream infections caused by CR-hvKP compared to the CR-cKP (Figure 3C).

Subgroup analysis based on patient department demonstrated notably high heterogeneity among studies focused on ICU populations (I^2^ = 79.6%), suggesting potential differences in baseline mortality risk or patient characteristics. Nevertheless, the ICU subgroup demonstrated a higher odds ratio than the non-ICU subgroup, indicating an increased mortality risk among patients in the ICU (Figure 3D).

### 3.5 Meta-regression

Meta-regression revealed no significant association between study strain collection year (median) and mortality effect size (coefficient = −0.05, *p* = 0.80; R^2^ = 0.0%), suggesting that temporal trends did not influence mortality outcomes across studies.

### 3.6 Sensitivity analysis and publication bias

**Figure 4:**
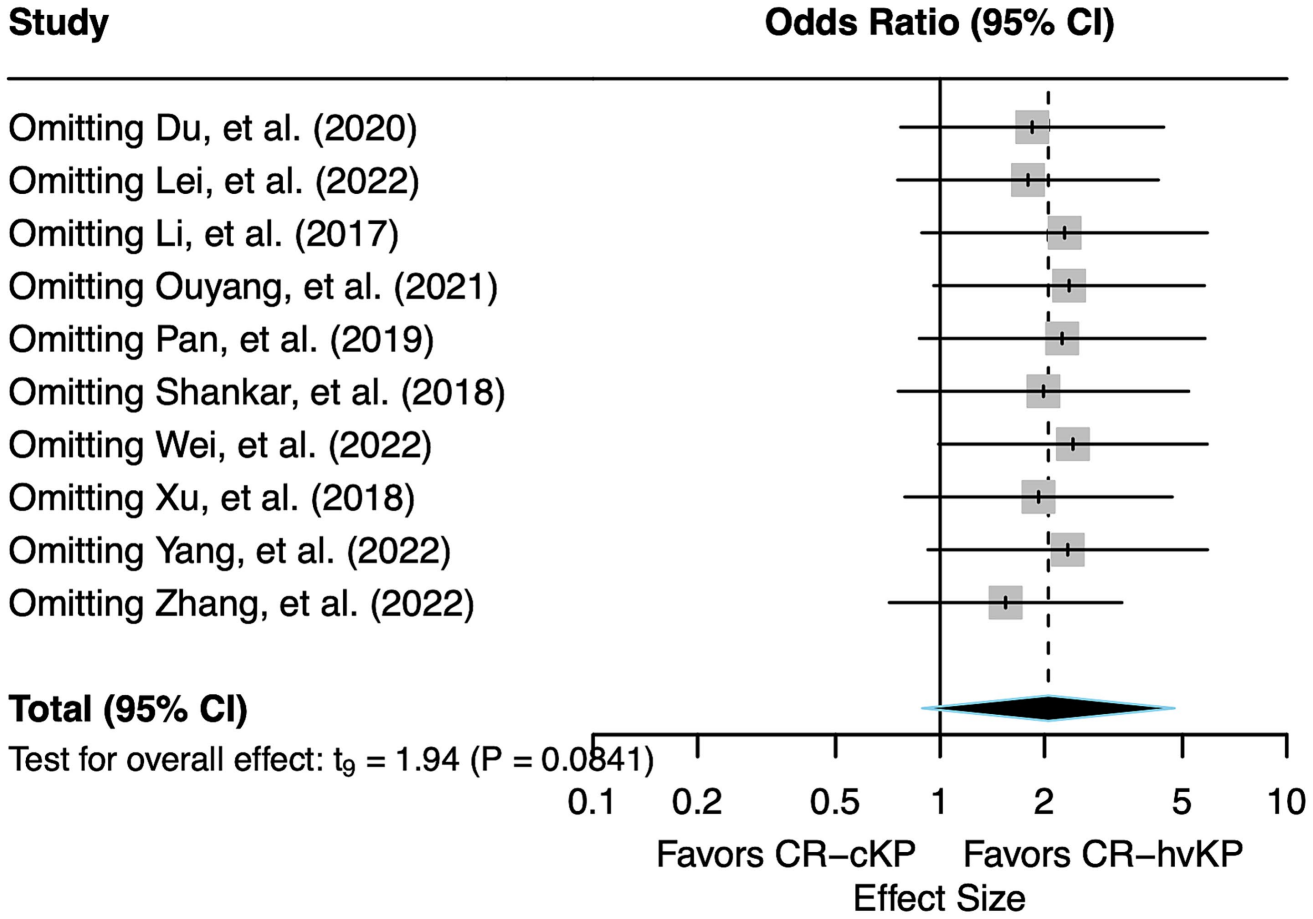
Forest plot of sensitivity analysis. Each study was sequentially removed to assess the robustness of the pooled effect size for mortality risk of CR-hvKP vs. CR-cKP.

Across all sensitivity analyses, the pooled effect consistently demonstrated an OR greater than 1, indicating a robust trend of increased mortality risk associated with CR-hvKP compared to CR-cKP. The observed heterogeneity remained moderate throughout (I^2^ = 50.0–69.9%), with no individual study exerting substantial influence on the overall between-study variation. Notably, all *p*-values exceeded the 0.05 significance threshold in these analyses, confirming that the observed associations maintained their non-significant status regardless of which study was excluded (Figure 4).

Taken together, the sensitivity analysis indicates that the overall findings of this meta-analysis are relatively robust and not overly dependent on any individual study. The effect estimates and heterogeneity measures remained broadly consistent, supporting the stability of the main results.

Funnel plot inspection revealed a relatively symmetrical distribution of effect estimates. Egger’s regression test indicated no statistically significant evidence of publication bias (t = 1.79, df = 8, *p* = 0.11) (Supplementary Figure S1).

## 4 Discussion

In this meta-analysis, we synthesized data from 10 studies comparing mortality outcomes between patients infected with CR-hvKP and CR-cKP. The pooled OR was 2.05 (95% CI: 0.89–4.75), indicating a non-significant trend toward higher mortality among patients with CR-hvKP infection compared to CR-cKP. While this trend suggests a possible increase in clinical severity, the lack of statistical significance warrants cautious interpretation. Moderate heterogeneity was observed across studies (I^2^ = 66.8%), prompting further stratified analyses to identify potential sources of variation.

Importantly, the most notable and robust finding of this study lies in the subgroup analysis demonstrating that phenotypic definitions of hypervirulence were associated with a significantly higher pooled OR (4.16) compared to genotypic definitions (1.05), with this difference reaching statistical significance (*p* < 0.05). This reduction in heterogeneity after stratification suggests that the method of hypervirulence detection is an important contributor to variability across studies. Notably, the string test, a phenotypic assay, may have greater distinguishing power in identifying truly pathogenic strains compared to genotypic approaches based solely on the presence of virulence genes. This difference likely reflects the fact that phenotypic tests capture the actual functional expression of hypermucoviscosity, which is a critical determinant of *in vivo* pathogenicity and clinical severity (18, 19). In contrast, detection of virulence genes alone does not guarantee their expression or activity, as gene regulation, plasmid carriage, and host–pathogen interactions can vary across strains. Therefore, phenotypic assays may provide a more direct link to clinical outcomes such as mortality, whereas genotypic screening may overestimate virulence potential if expression is absent or attenuated (20, 21). This underscores both the methodological heterogeneity in the field and the need for standardized, reliable definitions of hypervirulence in clinical research.

Subgroup analyses based on mortality timepoints provided additional insights into the prognostic evaluation of CR-hvKP infections. Among the included studies, all but one reported either 30-day or in-hospital mortality, with only a single study reporting 14-day mortality. The heterogeneity among studies reporting 30-day mortality was lower than that of studies reporting in-hospital mortality, suggesting that mortality outcomes measured at consistent and standardized timepoints yield more stable and reliable estimates. Moreover, although no statistically significant differences were observed between mortality timepoints (*p* > 0.05), the OR for 30-day mortality was lower than that for in-hospital mortality (1.42 vs. 2.46). In Zhang’s study, the median hospital stays among patients who died were 61.5 days, possibly reflecting the impact of CR-hvKP, which may lead to early clinical deterioration and prolonged hospitalization (22). Consequently, in-hospital mortality may encompass more late-phase deaths. These findings highlight the importance of selecting consistent and clinically meaningful mortality endpoints when assessing the outcomes of patients with CR-hvKP infections (23, 24).

The infection site also appeared to influence outcomes. Among patients with bloodstream infections, CR-hvKP was associated with increased mortality, with an OR of 4.05—higher than that observed in other infection types, suggesting that infection site may modulate the clinical consequences of hypervirulence in the setting of carbapenem resistance. Furthermore, subgroup analysis based on clinical department revealed substantial heterogeneity among ICU-based studies (I^2^ = 79.6%), indicating that differences in baseline patient severity, comorbidities, or care practices may confound the relationship between pathogen type and mortality. This highlights the need for future studies to account for patient-level factors and standardize inclusion criteria when comparing outcomes between CR-hvKP and CR-cKP. In addition, evaluation of strain virulence based on the median time point of sample collection failed to reveal a clear temporal trend, suggesting that temporal variation in strain acquisition does not adequately explain differences in mortality outcomes (R^2^ = 0.0%). Another important source of potential confounding arises from differences in antimicrobial therapy and source control. Clinical outcomes of patients with CR-hvKP and CR-cKP are likely to be strongly influenced by therapeutic strategies. For example, the availability of newer agents such as ceftazidime–avibactam may directly impact survival (25). However, none of the included studies provided a detailed comparison of therapeutic regimens between the two groups. Regarding underlying diseases, previous studies have demonstrated that diabetes mellitus and renal disease are risk factors for hvKP infection (26). In the included studies that reported relevant data, the distribution of diabetes and renal disease did not differ significantly between CR-hvKP and CR-cKP groups. In the study by Lei et al. (27), the proportion of patients older than 60 years was significantly higher in the hypervirulent group than in the classical group (*p* = 0.011). Wei et al. (28) reported a significant difference in the prevalence of cardiovascular disease between the two groups (*p* < 0.001). Nevertheless, given the limited number of available studies, no definitive conclusions can be drawn.

*K. pneumoniae* is often asymptomatically colonized in the gut, but hvKP can disseminate via the portal vein to the liver, leading to liver abscess formation (29). Liver abscess is considered an important clinical outcome of hvKP infection (4); however, only one included study explicitly reported this complication, showing no significant difference between the two groups (7.84% vs. 3.45%, *p* = 0.764). Likewise, effective source control interventions, such as abscess drainage or removal of infected devices, are critical determinants of prognosis. Lei et al. (27), Ouyang et al. (30), Wei et al. (28), and Yang et al. (31) described management of invasive procedures in both groups. Except for Wei et al. (28), which reported that gastric tube placement was significantly more frequent in patients with hypervirulent strains (*p* = 0.045), the other three studies found no significant differences in invasive medical procedures. However, most of the included studies did not provide detailed information on these factors, precluding formal adjustment. The absence of such data may bias comparisons of mortality between CR-hvKP and CR-cKP.

Sensitivity analyses confirmed the robustness of the primary findings. Across all iterations, the direction of the effect remained consistent, and no single study disproportionately influenced the overall estimates. Nevertheless, several limitations warrant consideration. Most notably, nearly all included studies originated from China, which may limit the generalizability of our findings to other geographic regions. In China, CR-hvKP is predominantly associated with the ST11 clone, particularly ST11-K64 and ST11-K47 lineages, which frequently harbor conjugative plasmids carrying both carbapenemase genes and virulence determinants (32–35). This convergence of resistance and virulence within a single epidemic lineage may partly explain the consistently elevated mortality observed in Chinese cohorts. By contrast, molecular epidemiology varies across regions: ST147 and ST395 have been reported as prominent clones in the United Kingdom and Russia, respectively, while in Southeast Asia, Europe, and the Americas, hvKP is more often linked to sequence types such as ST23, ST65, or ST86 (11, 36–38). These geographic differences in dominant lineages and their resistance-virulence balance may influence clinical outcomes. As such, our pooled estimates largely reflect the Chinese molecular-epidemiological context, and readers should exercise caution when extrapolating these results to non-Asian populations or settings with distinct lineage distributions. Future multicenter studies from diverse regions are needed to validate whether the observed associations hold across different molecular backgrounds. Additionally, the relatively small number of eligible studies and the wide confidence intervals reduce the statistical power and precision of the pooled estimates. Another important limitation lies in the restricted availability of individual-level data. Due to limited reporting in the original studies, we were unable to systematically assess baseline patient characteristics such as age, comorbidities, immunosuppression status, and disease severity factors. Therefore, unmeasured confounding related to baseline clinical conditions or therapeutic management cannot be excluded, and this residual bias may partially account for variability in observed mortality risk.

Moreover, information regarding treatment regimens, including antimicrobial choices, source control strategies, and timing of intervention, was either inconsistently reported or absent, hindering our ability to evaluate the role of management differences in patient prognosis. Given the emergence of novel agents such as ceftazidime–avibactam and meropenem-vaborbactam, differences in therapeutic availability or clinical practice may substantially influence patient outcomes. Finally, this meta-analysis focused solely on mortality, and other clinically relevant outcomes such as ICU admission rate, incidence of organ failure (e.g., renal dysfunction), length of hospital stay, and recurrence of infection were not assessed due to insufficient data. These outcomes are crucial for assessing the comprehensive clinical burden of CR-hvKP infections and should be incorporated into future research.

## 5 Conclusion

**Figure 5:**
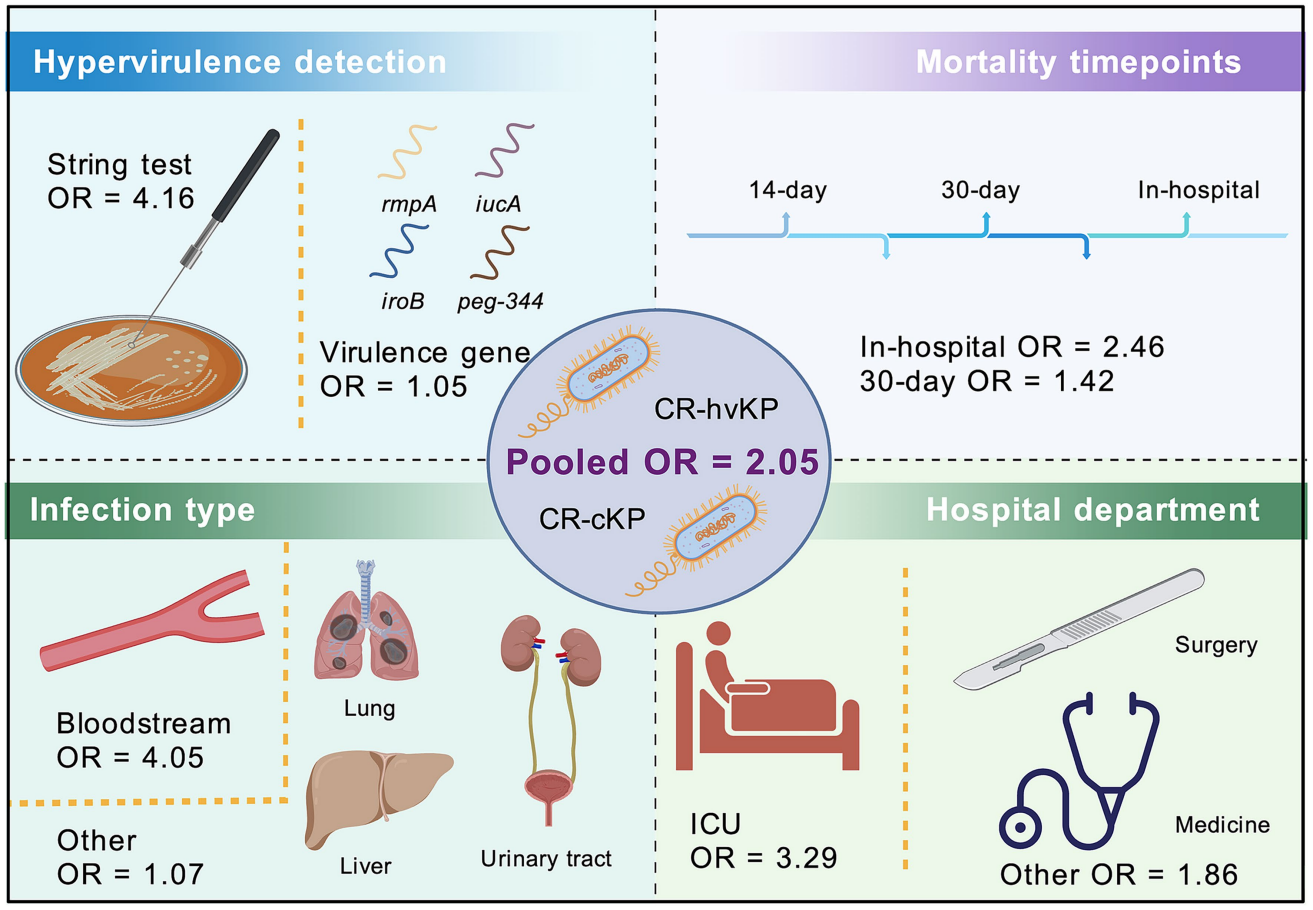
Schematic illustration of the meta-analysis comparing mortality risk between CR-hvKP and CR-cKP. The OR for mortality is illustrated as 2.05 for CR-hvKP compared to CR-cKP. Subgroup analyses were conducted based on the definition of hypervirulence (string test vs. virulence gene), mortality timepoints (in-hospital vs. 30-day), infection type bloodstream vs. other, and hospital department (ICU vs. other). Icons represent key elements of the analysis, including patient source, bacterial strains, and influencing clinical factors.

In summary, our results indicate a non-significant trend toward higher mortality associated with CR-hvKP infections. Nevertheless, the definition of hypervirulence emerged as the key determinant of effect size, with phenotypic criteria showing a significantly higher mortality association than genotypic definitions. However, considerable variability across studies—in terms of definition criteria, infection types, and patient departments—highlights the need for standardized definitions of hypervirulence and prospective, multicenter studies to better understand the clinical implications of CR-hvKP (Figure 5). The stronger effect associated with phenotypically defined hvKP may reflect true biological differences or misclassification bias arising from genotypic-only definitions.

## Data Availability

The original contributions presented in the study are included in the article/Supplementary material, further inquiries can be directed to the corresponding authors.

## References

[1] Paczosa MK Mecsas J. *Klebsiella Pneumoniae*: going on the offense with a strong defense. Microbiol Mol Biol Rev. (2016) 80:629–61. doi: 10.1128/mmbr.00078-15, PMID: 27307579 PMC4981674

[2] Yang X Wai-Chi Chan E Zhang R Chen S. A conjugative plasmid that augments virulence in *Klebsiella Pneumoniae*. Nat Microbiol. (2019) 4:2039–43. doi: 10.1038/s41564-019-0566-7, PMID: 31570866

[3] Liu YC Cheng DL Lin CL. *Klebsiella pneumoniae* liver abscess associated with septic endophthalmitis. Arch Intern Med. (1986) 146:1913–6. 3532983

[4] Nagendra D R N Shanbhag V Eshwara VK Ramamoorthi K Priya PS . Incidental detection of hypervirulent *Klebsiella pneumoniae* liver abscess with systemic and cardiac complications in an elderly diabetic woman. Radiol Case Rep. (2025) 20:4674–8. doi: 10.1016/j.radcr.2025.06.033, PMID: 40677891 PMC12268016

[5] Oka K Yamamoto N Amano S Sano C Ohta R. Recurrent dissemination despite local control in a case of Hypervirulent *Klebsiella pneumoniae* liver abscess: a case report. Cureus. (2025) 17:e85438. doi: 10.7759/cureus.85438, PMID: 40621340 PMC12228582

[6] Baronos K Scott S Hebbes C. Disseminated Hypervirulent *Klebsiella Pneumoniae* infection following travel: a case of Cavitating pneumonia, hepatic and renal abscesses, and thrombosis. Cureus. (2025) 17:e82059. doi: 10.7759/cureus.82059, PMID: 40351917 PMC12066016

[7] Melot B Brisse S Breurec S Passet V Malpote E Lamaury I . Community-acquired meningitis caused by a Cg86 Hypervirulent *Klebsiella Pneumoniae* strain: first case report in the Caribbean. BMC Infect Dis. (2016) 16:736. doi: 10.1186/s12879-016-2065-2, PMID: 27923372 PMC5142283

[8] Wang W Tian D Hu D Chen W Zhou Y Jiang X. Different regulatory mechanisms of the capsule in Hypervirulent Klebsiella pneumonia: "direct" Wcaj variation vs. "indirect" Rmpa regulation. Front Cell Infect Microbiol. (2023) 13:1108818. doi: 10.3389/fcimb.2023.1108818 37180440 PMC10168181

[9] Rocha J Henriques I Gomila M Manaia CM. Common and distinctive genomic features of *Klebsiella Pneumoniae* thriving in the natural environment or in clinical settings. Sci Rep. (2022) 12:10441. doi: 10.1038/s41598-022-14547-6, PMID: 35729190 PMC9213442

[10] Gorrie CL Mirčeta M Wick RR Judd LM Lam MMC Gomi R . Genomic dissection of *Klebsiella Pneumoniae* infections in hospital patients reveals insights into an opportunistic pathogen. Nat Commun. (2022) 13:3017. doi: 10.1038/s41467-022-30717-6, PMID: 35641522 PMC9156735

[11] Wyres KL Lam MMC Holt KE. Population genomics of *Klebsiella Pneumoniae*. Nat Rev Microbiol. (2020) 18:344–59. doi: 10.1038/s41579-019-0315-1, PMID: 32055025

[12] Cao Z Meng B Chen J Ge R Liu Y Li J. β-Nicotinamide mononucleotide protects against hypervirulent *Klebsiella pneumoniae* bloodstream infection and liver injury. mSphere. (2025) 10:e0036125. doi: 10.1128/msphere.00361-25 40742124 PMC12379607

[13] Pu D Zhao J Chang K Zhuo X Cao B. "superbugs" with Hypervirulence and Carbapenem resistance in *Klebsiella Pneumoniae*: the rise of such emerging nosocomial pathogens in China. Sci Bull. (2023) 68:2658–70. doi: 10.1016/j.scib.2023.09.040, PMID: 37821268

[14] Li R Liu J Yang L Lin Z Rong L Chen G . Pulmonary abscess secondary to epididymitis caused by extended Spectrum β-lactamase-producing Hypervirulent *Klebsiella Pneumoniae*: a case report. BMC Infect Dis. (2024) 24:820. doi: 10.1186/s12879-024-09721-2, PMID: 39138429 PMC11321085

[15] García-Cobos S Oteo-Iglesias J Pérez-Vázquez M. Hypervirulent *Klebsiella pneumoniae*: epidemiology outside Asian countries, antibiotic resistance association, methods of detection and clinical management. Enfermedades Infecciosas Microbiol Clin. (2025) 43:102–9. doi: 10.1016/j.eimce.2024.12.008, PMID: 39914938

[16] Wang X Wang J Jiang X Huang Z Huang L Wei Q . Molecular epidemiological analysis and research on resistance and virulence of Carbapenem-resistant *Klebsiella Pneumoniae* in a tertiary hospital from 2016 to 2023. BMC Microbiol. (2025) 25:217. doi: 10.1186/s12866-025-03888-7, PMID: 40234763 PMC12001477

[17] Liu Y Wang Z Jian Z Liu P Li Y Qin F . Nosocomial transmission, adaption and clinical outcomes of Carbapenem-resistant Hypervirulent *klebsiella Pneumoniae*. BMC Microbiol. (2025) 25:376. doi: 10.1186/s12866-025-04096-z, PMID: 40597682 PMC12219914

[18] Hyun M Lee JY Kim HA. Clinical and microbiologic analysis of *Klebsiella Pneumoniae* infection: Hypermucoviscosity, virulence factor, genotype, and antimicrobial susceptibility. Diagnostics. (2024) 14:792. doi: 10.3390/diagnostics14080792 38667438 PMC11048833

[19] Russo TA Lebreton F McGann PT. A step forward in Hypervirulent *Klebsiella Pneumoniae* diagnostics. Emerg Infect Dis. (2025) 31:1–3. doi: 10.3201/eid3101.241516 PMC11682795 39714290

[20] Shankar C Nabarro LE Anandan S Ravi R Babu P Munusamy E . Extremely high mortality rates in patients with Carbapenem-resistant, Hypermucoviscous *Klebsiella Pneumoniae* blood stream infections. J Assoc Physicians India. (2018) 66:13–6. 31313543

[21] Al Ismail D Campos-Madueno EI Donà V Endimiani A. Hypervirulent *Klebsiella Pneumoniae* (Hvkp): overview, epidemiology, and laboratory detection. Pathog Immun. (2024) 10:80–119. doi: 10.20411/pai.v10i1.777, PMID: 39911145 PMC11792540

[22] Zhang N Qi L Liu X Jin M Jin Y Yang X . Clinical and molecular characterizations of Carbapenem-resistant *Klebsiella pneumoniae* causing bloodstream infection in a Chinese hospital. Microbiol Spectr. (2022) 10:e0169022. doi: 10.1128/spectrum.01690-22, PMID: 36190403 PMC9603270

[23] Liao W Zhao T Zhang Z Yan F Peng X Cui J . Fatal stent-associated respiratory tract infection caused by K64-St11 Kpc-2-producing carbapenem-resistant hypervirulent *Klebsiella pneumoniae*: a rare case report. Microb Drug Resist. (2023) 29:28–33. doi: 10.1089/mdr.2022.0193, PMID: 36656990

[24] Cai Z Jia T Pu M Zhang S Zhang J Geng R . Clinical and molecular analysis of St11-K47 Carbapenem-resistant Hypervirulent *Klebsiella Pneumoniae*: a strain causing liver abscess. Pathogens. (2022) 11:657. doi: 10.3390/pathogens11060657, PMID: 35745510 PMC9227846

[25] Ishag MY Alsuleimani AL. Invasive *Klebsiella Pneumoniae* causing concurrent liver and pulmonary abscesses: successful management with prolonged Oral amoxicillin-Clavulanate. Cureus. (2025) 17:e87412. doi: 10.7759/cureus.87412, PMID: 40772223 PMC12327917

[26] Najafian H Pouresmaeil O Meshkat Z Aryan E Falahi J Najafian L . Determination of the prevalence of hypervirulent *Klebsiella pneumoniae* strains in Northeast Iran, Mashhad. Sci Rep. (2025) 15:31579. doi: 10.1038/s41598-025-16969-4, PMID: 40866467 PMC12391358

[27] Lei J Zhou WX Lei K Chen D Zhang PQ Xue L . Analysis of molecular and clinical characteristics of carbapenem-resistant hypervirulent *Klebsiella pneumoniae* in the intensive care unit. Chin J Prev Med. (2022) 56:63–8. doi: 10.3760/cma.j.cn112150-20210812-00781, PMID: 35092993

[28] Wei T Zou C Qin J Tao J Yan L Wang J . Emergence of Hypervirulent St11-K64 *Klebsiella Pneumoniae* poses a serious clinical threat in older patients. Front Public Health. (2022) 10:765624. doi: 10.3389/fpubh.2022.765624, PMID: 35309213 PMC8930914

[29] Angeles-Solano M Tabashsum Z Chen L Rowe SE. *Klebsiella Pneumoniae* liver abscesses: pathogenesis, treatment, and ongoing challenges. Infect Immun. (2025) 93:e0050824. doi: 10.1128/iai.00508-24, PMID: 40607938 PMC12341377

[30] Ouyang P Jiang B Peng N Wang J Cai L Wu Y . Characteristics of St11 Kpc-2-producing Carbapenem-resistant Hypervirulent *Klebsiella Pneumoniae* causing nosocomial infection in a Chinese hospital. J Clin Lab Anal. (2022) 36:e24476. doi: 10.1002/jcla.24476, PMID: 35522153 PMC9169163

[31] Yang P Wu Z Liu C Zheng J Wu N Wu Z . Clinical outcomes and microbiological characteristics of sequence type 11 *Klebsiella pneumoniae* infection. Front Med Lausanne. (2022) 9:889020. doi: 10.3389/fmed.2022.889020, PMID: 35652076 PMC9149164

[32] Cao H Liang S Zhang C Liu B Fei Y. Molecular profiling of a multi-strain Hypervirulent *Klebsiella Pneumoniae* infection within a single patient. Infect Drug Resist. (2023) 16:1367–80. doi: 10.2147/idr.S404202, PMID: 36937147 PMC10017834

[33] Fang J Lai H Deng M Mei Y Chen D Hou T . Mechanism of virulence polymorphism in Cr-Hvkp strains from the same source. Microbiol Spectr. (2025) 13:e0246424. doi: 10.1128/spectrum.02464-24, PMID: 40407310 PMC12210850

[34] Li L Liang J Zhang H Guo J Li S Li M. Emergence and clinical challenges of St11-K64 Carbapenem-resistant *Klebsiella Pneumoniae*: molecular insights and implications for antimicrobial resistance and virulence in Southwest China. BMC Infect Dis. (2025) 25:19. doi: 10.1186/s12879-024-10390-4, PMID: 39754049 PMC11699810

[35] Shi Q Shen S Tang C Ding L Guo Y Yang Y . Molecular mechanisms responsible Kpc-135-mediated resistance to Ceftazidime-avibactam in St11-K47 Hypervirulent *Klebsiella Pneumoniae*. Emerg Microbes Infect. (2024) 13:2361007. doi: 10.1080/22221751.2024.2361007, PMID: 38801099 PMC11172257

[36] Sohrabi M Alizade Naini M Rasekhi A Oloomi M Moradhaseli F Ayoub A . Emergence of K1 St23 and K2 St65 Hypervirulent *Klebsiella Pneumoniae* as true pathogens with specific virulence genes in cryptogenic pyogenic liver abscesses shiraz Iran. Front Cell Infect Microbiol. (2022) 12:964290. doi: 10.3389/fcimb.2022.964290, PMID: 36017366 PMC9396702

[37] Maguire M DeLappe N Clarke C Touhy A Carlino-MacDonald U Hutson A . Genomic and phylogenetic analysis of Hypervirulent *Klebsiella Pneumoniae* St23 in Ireland. Microb Genom. (2025) 11:373. doi: 10.1099/mgen.0.001373, PMID: 40106330 PMC12452180

[38] McElheny CL Iovleva A Chen N Woods D Pradhan A Sonnabend JL . Prevalence and features of hypervirulent *Klebsiella pneumoniae* in respiratory specimens at a us hospital system. Infect Immun. (2025) 93:e0048624. doi: 10.1128/iai.00486-24 39660916 PMC11784238

[39] Du F Wei D Mei Y Long D Wan L Liu Y. Clinical and molecular characteristics of Carbapenem-resistant Hypermucoviscous *Klebsiella Pneumoniae* isolated from bloodstream infection in Icu patients. Chin J Infect Chemother. (2020) 20:181–6. doi: 10.16718/j.1009-7708.2020.02.013

[40] Li J Ren J Wang W Wang G Gu G Wu X . Risk factors and clinical outcomes of Hypervirulent *Klebsiella Pneumoniae* induced bloodstream infections. Eur J Clin Microbiol. (2018) 37:679–89. doi: 10.1007/s10096-017-3160-z, PMID: 29238932

[41] Pan H Lou Y Zeng L Wang L Zhang J Yu W . Infections caused by carbapenemase-producing *Klebsiella pneumoniae*: microbiological characteristics and risk factors. Microb Drug Resist. (2019) 25:287–96. doi: 10.1089/mdr.2018.0339, PMID: 30810470 PMC6441289

[42] Xu M Fu Y Kong H Chen X Chen Y Li L . Bloodstream infections caused by *Klebsiella Pneumoniae*: prevalence of Bla(Kpc), virulence factors and their impacts on clinical outcome. BMC Infect Dis. (2018) 18:358. doi: 10.1186/s12879-018-3263-x, PMID: 30064360 PMC6069789

