# Supplementary material for: Mortality risk of carbapenem-resistant hypervirulent Klebsiella pneumoniae vs. classical CR-KP: a systematic review and meta-analysis
Source: Front Public Health. 2025 Nov 6;13:1680292. doi: 10.3389/fpubh.2025.1680292 (PMC12631208; doi:10.3389/fpubh.2025.1680292)
Supplement: Supplementary file 2 [file Data_Sheet_1.docx]

Supplementary Material

# Supplementary Figures and Tables

## Supplementary Figures


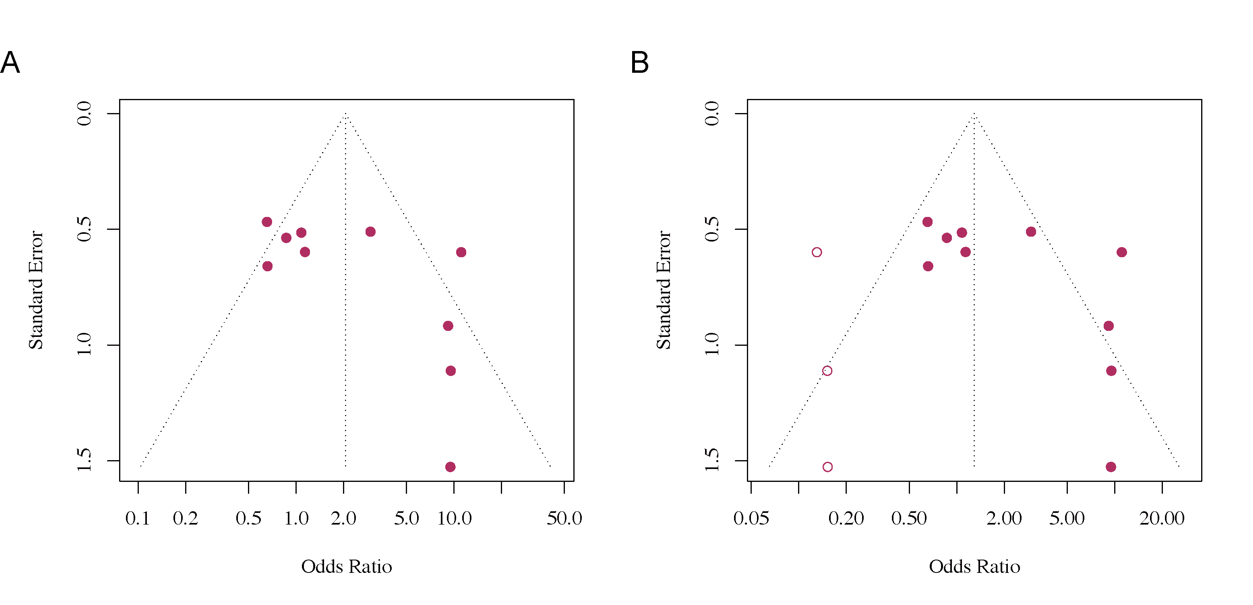


**Supplementary Figure 1.** Funnel plot of the studies included in the meta-analysis assessing publication bias. (a) Funnel plot, (b) Trim and Fill funnel plot.

## Supplementary Tables

**Supplementary Table S1.** Detailed Search Strategies for Each Database

**Supplementary Table S2.** Source Information Extracted from Included Studies.

**Supplementary Table S3.** Quality Assessment Scores of Included Studies.
